# Alginate Microencapsulated Hepatocytes Optimised for Transplantation in Acute Liver Failure

**DOI:** 10.1371/journal.pone.0113609

**Published:** 2014-12-01

**Authors:** Suttiruk Jitraruch, Anil Dhawan, Robin D. Hughes, Celine Filippi, Daniel Soong, Christina Philippeos, Sharon C. Lehec, Nigel D. Heaton, Maria S. Longhi, Ragai R. Mitry

**Affiliations:** 1 Institute of Liver Studies, King's College London School of Medicine, London, United Kingdom; 2 British Heart Foundation Centre of Excellence Cardiovascular Division, King's College London School of Medicine, London, United Kingdom; UNIFESP Federal University of São Paulo, Brazil

## Abstract

**Background and Aim:**

Intraperitoneal transplantation of alginate-microencapsulated human hepatocytes is an attractive option for the management of acute liver failure (ALF) providing short-term support to allow native liver regeneration. The main aim of this study was to establish an optimised protocol for production of alginate-encapsulated human hepatocytes and evaluate their suitability for clinical use.

**Methods:**

Human hepatocyte microbeads (HMBs) were prepared using sterile GMP grade materials. We determined physical stability, cell viability, and hepatocyte metabolic function of HMBs using different polymerisation times and cell densities. The immune activation of peripheral blood mononuclear cells (PBMCs) after co-culture with HMBs was studied. Rats with ALF induced by galactosamine were transplanted intraperitoneally with rat hepatocyte microbeads (RMBs) produced using a similar optimised protocol. Survival rate and biochemical profiles were determined. Retrieved microbeads were evaluated for morphology and functionality.

**Results:**

The optimised HMBs were of uniform size (583.5±3.3 µm) and mechanically stable using 15 min polymerisation time compared to 10 min and 20 min (p<0.001). 3D confocal microscopy images demonstrated that hepatocytes with similar cell viability were evenly distributed within HMBs. Cell density of 3.5×10^6^ cells/ml provided the highest viability. HMBs incubated in human ascitic fluid showed better cell viability and function than controls. There was no significant activation of PBMCs co-cultured with empty or hepatocyte microbeads, compared to PBMCs alone. Intraperitoneal transplantation of RMBs was safe and significantly improved the severity of liver damage compared to control groups (empty microbeads and medium alone; p<0.01). Retrieved RMBs were intact and free of immune cell adherence and contained viable hepatocytes with preserved function.

**Conclusion:**

An optimised protocol to produce GMP grade alginate-encapsulated human hepatocytes has been established. Transplantation of microbeads provided effective metabolic function in ALF. These high quality HMBs should be suitable for use in clinical transplantation.

## Introduction

Acute liver failure (ALF) is a devastating condition which causes an abrupt loss of hepatic function leading to encephalopathy, coagulopathy and progressive multiple organ failure. The mortality of ALF is high without orthotopic liver transplantation [1]. Liver transplantation is an effective treatment but suffers from following limitations scarcity of organ donors, surgical risk, and requirement for life-long immunosuppression. Intrahepatic hepatocyte transplantation has shown benefit as a bridge to transplantation [2], [3]. However, invasive catheter placement in the liver in a coagulopathic patient and use of immunosuppression are perceived as high risk factors. Hence a technique that would avoid use of immunosuppression and transplantation of cells in a body cavity that easily accessible will be an ideal scenario. Alginate encapsulated microencapsulated hepatocytes seems to be the best fit [4], [5]. The principle of the microencapsulation technique is that the cells are embedded in a semi-permeable polymerised structure with the aim of protecting cells from host immune attack, while permitting the diffusion of nutrients, oxygen and metabolic products which maintain cell survival and function [6]. Thus this approach allows cell transplantation without using immunosuppression and also avoids the risk of bleeding. Microencapsulation could also protect hepatocytes from cryoinjury leading to improved cell viability and function [7], [8], allowing cryopreserved cells to be available for emergency transplantation in ALF patients.

Microbeads need to maintain their integrity during transplantation and within the implant site, as damage would result in functional loss of cells and immune rejection [9]. The mechanical stability can be optimised by factors such as time of cross-linking (polymerisation), and spatial distribution of cells. However, increasing the strength of microbeads could reduce permeability which may undermine cell viability and function, therefore, these properties need to be optimised [10].

The biomaterial of the microbeads, or antigens/chemokines released through the pores of the microbeads from encapsulated cells, may initiate a host immune response and subsequently lead to an inflammatory reaction and cell death [11]. The degree of alginate purity has been shown to be of great importance in both *in vitro* and *in vivo* studies [12]. Our main aim was to produce high quality microencapsulated hepatocytes using materials of good manufacturing practice (GMP) grade for clinical use.

In this study we optimised the microbeads by studying the effects of polymerisation time and cell density on physical integrity and hepatocyte-specific functions *in vitro*. We investigated transplantation of rat hepatocyte microbeads *in vivo* to evaluate the efficacy and safety of transplantation in a rat model of ALF using D-galactosamine (D-GalN) administration.

## Materials and Methods

### Ethics statements

All human tissues were approved for research use in accordance with the Research Ethics Committee of King's College Hospital. Written informed consent was obtained from donor relatives or patients.

All animal experiments were performed following protocols approved by the Ethical Review Process of King's College London in accordance with the UK Animals (Scientific) Procedures Act of 1986.

### Hepatocyte isolation

#### Human hepatocyte isolation

Human hepatocytes were isolated from donor liver tissues (rejected or unused for transplantation) or from the non-tumoral margin of liver resections from metastatic cancer cases using a collagenase perfusion technique according to Mitry (2009) [13]. Total hepatocyte number and their viability were estimated using the standard trypan blue exclusion test; cell viability was ≥60%. In some experiments cryopreserved human hepatocytes were used [14].

#### Rat hepatocyte isolation

Rat hepatocyte isolation was performed using *in situ* collagenase perfusion of the liver as previously published [15], [16]. Isolated hepatocytes were purified by three times low speed centrifugation (50xg, 4°C for 5 min) and washed with ice-cold EMEM. The total number of hepatocytes and viability were determined using the standard trypan blue exclusion test.

### Microencapsulation of hepatocytes (hepatocyte microbeads)

#### Human hepatocyte microbeads

Empty and human hepatocyte microbeads (EMBs and HMBs) were produced using the IE-50R encapsulator (Inotech Encapsulation AG, Dottikon, Switzerland), and sterile clinical grade and GMP materials. Ultra-pure sodium alginate, with low viscosity and high glucoronic acid (PRONOVA SLG20; NovaMatrix, Sandvika, Norway) was dissolved in 0.9% NaCl to give a final concentration of 1.5% alginate solution (w/v). HMBs were produced using a 250 µm nozzle, and polymerised in 1.2% CaCl_2_ solution. The microbeads were washed twice with 0.9% NaCl to remove excess Ca^2+^ ions.

#### Rat hepatocyte microbeads

Rat hepatocyte microbeads (RMBs) were prepared using the optimised protocol that has been established in this study. Preliminary studies had shown that this protocol was also optimal for rat hepatocytes. After production, microbeads were allocated for *in vitro* study and transplantation. RMBs were maintained in CMRL prior to transplantation.

### Hepatocyte microbeads culture

HMBs or RMBs were re-suspended in culture medium consisting of CMRL (Mediatech, Inc., Vancouver, Canada) supplemented with 10% heat-inactivated FCS, 2 mM L-glutamine, 0.1 µM dexamethasone, 0.1 µM insulin (Sigma-Aldrich, Gilingham, UK), and penicillin (50 U/ml) and streptomycin (50 µg/ml, Gibco, Paisley, UK). Microbeads were maintained in this culture medium at a ratio of 1∶4 (microbeads: medium; v:v) in a humidified incubator at 37°C and 5% CO_2_ for 3 days. Supernatants were changed every alternate day and were collected at each time point according to the experimental setting (day 1, 2, 3 and 7) for analysis.

### Cell membrane integrity assay and overall metabolic activity assays

Microbeads (100 µl) were washed with Dulbecco's phosphate-buffered saline (DPBS; Gibco), then resuspended in 1 ml DPBS containing 10 µg fluorescein diacetate (FDA; Sigma-Aldrich) and 20 µg propidium iodide (PI; Sigma-Aldrich), and incubated in the dark for 90 sec at room temperature (RT). Microbeads were washed three times with DPBS followed by adding VECTASHIELD Mounting Media (Vector Laboratories Ltd., Peterborough, UK) then visualised under a fluorescent microscope (Leica Microsystems Ltd, Milton Keynes, UK). Live cell cytoplasm appeared bright green (FDA), while dead cells had bright red-stained nuclei (PI). The overall metabolic activity of encapsulated hepatocytes was evaluated by measuring the reduction of tetrazolium salt [3-(4, 5-dimethylthiazol-2-yl)-2,-5 diphenyltetrazolium bromide; MTT], [17]. The values were expressed as mean OD reading±SEM per 100 mg microbeads.

### Hepatocyte-specific functions

The amount of albumin secreted by HMBs and RMBs over 24 h was quantified using the Human and Rat Albumin sandwich enzyme-linked-immunosorbent-assay (ELISA) Quantitation Kit (Bethyl Laboratories, Tx, USA), respectively. Urea synthesis was assessed after challenging hepatocytes with ammonium chloride (5 mM) for 6 h at 37°C. Supernatant was collected and analysed using the QuantiChrom Urea Assay Kit (BioAssay Systems, CA, USA). Cytochrome P450 (CYP1A1/2) activity was measured using the ethoxyresorufin O-deethylase (EROD) method [18]. Fluorescence was measured at 530 nm excitation/590 nm emission using a Tecan Genios Pro plate reader (Tecan Group Ltd., Männedrof, Switzerland). Total amount of resorufin produced was calculated using a resorufin standard curve (0–800 pmol).

### Assessment of physical integrity of microbeads-osmotic pressure test

HMBs were produced using 3 different polymerisation times (10, 15, and 20 min). Physical stability of microbeads was determined using an osmotic stress test. To establish the stability of microbeads preliminary experiments were done on EMBs. Based on these results, the integrity of HMBs was determined in CMRL (isotonic solution) and water (hypotonic solution). HMBs were assessed after 3 h of exposure to osmotic shock by measuring the diameter of 100 microbeads per sample under a light microscope, and using Image J software (ver. 1.44p, National Institutes of Health, USA). Microbeads immediately after production were used as controls.

### Effect of polymerisation time on hepatocyte viability and function in HMBs

To study the effect of polymerisation time in CaCl_2_ on hepatocyte viability and function, three polymerisation times (10, 15, and 20 min) were used in the production of HMBs. Samples of HMBs were exposed to the osmotic stress test followed by MTT assay to assess overall cell viability. Other HMBs samples were maintained in culture supplemented in CMRL medium for 24 h, followed by assessment of cell viability and hepatocyte-specific functions.

### Effects of cell density on microbead morphology, cell viability and function

HMBs were produced using 4 cell densities: 2.0, 2.5, 3.0 and 3.5 million cells/ml alginate using the same batch of cryopreserved human hepatocytes for each experiment. Cell density of 4.0×10^6^ cells/ml alginate was also studied in preliminary experiments, however, it was observed that the HMBs obtained varied in size (range: 400-1200 µm), and some had a distorted shape. Microbead morphology was examined immediately after production (control). HMBs were maintained in culture in complete CMRL for 3 days, and cell viability was assessed at day1.

Confocal microscopy was used to reconstruct 3D images of the microbeads. Briefly, 250 µl HMBs were stained with FDA/PI in DPBS. VECTASHIELD Mounting Media was added to prevent photo bleaching. HMBs were loaded onto glass slide with a well followed by addition of a drop of glycerol to minimise microbeads movement during imaging. The images were captured using a Leica SP5 confocal microscope (Leica Microsystems Ltd, Milton Keynes, UK). Images were imported into Volocity software (Perkin Elmer, Cambridge, UK) and then into Mathematica 8.0 (Wolfram Inc.,Oxfordsire, UK). This technique determined cell viability in relation to cell distribution in the microbeads (outer and inner halves), and the average number of cells per microbead. Hepatocyte-specific functions were also evaluated on day 1 and day 3.

### Effects of ascitic fluid on HMBs

Ascitic fluid was obtained from the abdominal drains of paediatric patients with ALF. HMBs were incubated in 100%, 50%, 25% ascitic fluid diluted with culture medium, and full culture medium (control). Cell viability and urea synthesis of encapsulated hepatocytes were assessed at days 1, 2, 3 and 7. It was difficult to measure albumin as the level in the ascitic fluid was already high.

### Microbeads immunogenicity

Peripheral blood mononuclear cells (PBMCs) were isolated from blood of four normal healthy donors. The isolated PBMCs were either cultured alone (controls) or co-cultured with EMBs or HMBs (3.5×10^6^ cells/ml alginate) in WEM with supplements. The cultures were maintained in a humidified incubator at 37°C for 24 h. The ratio of PBMCs to hepatocytes in co-cultures was 1∶5, based on preliminary experiments of direct co-culture of PBMCs and plated hepatocytes. Microbeads before and after co-culture with PBMCs were examined under a light microscope to evaluate the extent of cell adhesion to their surface in accordance to Tam et al (2011), [19]. Measurements of activated PBMCs following co-culture with either EMBs or HMBs were performed using three-color flow cytometry (FACS) analysis [20] in the presence of monoclonal antibodies to CD3, CD4, CD8, CD25, CD14, CD40L, CD38, CD56, CD19, and CD54 according to the combinations in Table 1.

**Table 1:** Percentage of frequency of activation markers expressed on PBMCs.

Activation marker	PBMCs alone	PBMCs after co-culture with EMBs	PBMCs after co-culture with HMBs
**CD3^+^ CD25^+^**	9.70±2.07	10.30±2.55	9.19±1.70
**CD4^+^ CD25^+^**	3.74±0.69	4.32±0.94	4.83±1.07
**CD8^+^ CD25^+^**	2.36±0.62	1.85±0.51	1.65±0.32
**CD14^+^ CD25^+^**	45.65±10.98	22.76±7.96	2.66±0.80*
**CD14 ^+^CD40L^+^**	6.39±1.34	5.32±1.95	6.36±2.32
**CD3^+^ CD54^+^**	14.28±2.98	13.38±1.04	11.36±2.11
**CD4^+^ CD54^+^**	2.53±0.73	4.17±1.17	1.90±0.69
**CD3^+^ CD56^+^**	12.75±3.17	12.10±1.32	9.40±1.48
**CD8^+^ CD54^+^**	13.54±3.52	12.26±1.31	12.64±2.04
**CD3^+^ CD38^+^**	6.11±1.14	6.78±0.68	6.75±0.89
**CD4^+^ CD38^+^**	2.72±0.16	3.22±0.55	2.12±0.34
**CD8^+^ CD38^+^**	10.03±4.99	11.77±5.63	9.90±4.23
**CD19^+^ CD25^+^**	0.61±0.24	1.07±0.29	2.55±1.04
**CD19^+^ CD54^+^**	1.10±0.28	2.30±0.50	5.54±2.14

Significance compared to control *p<0.001.

### *In vivo* experiments

#### Animals

Sprague Dawley (Harlan Olec, Bicester, UK), male rats 8–10 weeks old and weight between 200 to 300 g were used as the donor to isolate hepatocytes and recipients of hepatocyte microbeads. Animals were maintained in conventional housing facilities and received standard care. They were housed in room kept temperature at 21±2°C, humidity of 55±10% and 12-hour light-dark cycle with ad libitum food and water. The experiments were conducted after acclimatization for 7 days.

#### Induction of acute liver failure

Acute liver failure was induced with D-GalN. Briefly, D-GalN was freshly prepared at the time of use by dissolving in dH_2_O to a final concentration of 240 mg/ml and adjusted to physiological pH (7.4) using 5 M NaOH. Each rat received a single intraperitoneal injection of D-GalN at the dose of 1.2 g/kg. After injection, all animals were allowed a standard diet and 5% dextrose water ad libitum to prevent hypoglycemia.

#### Intraperitoneal transplantation of rat hepatocyte microbeads and experimental groups

Microbeads were transplanted into the peritoneal cavity of ALF animals at 24–28 h after D-GalN injection. The microbeads were suspended in transplant medium (CMRL) and were transplanted intraperitoneally via an 18-guage intravascular catheter into the rats using sterile technique. Each animal received microbeads suspension at a dose of 10 ml/kg, corresponding to approximately 8.75×10^6^ to 10.5×10^6^ cells per rat. Animals were anesthetised with isoflurane for the procedure. The animals were allocated to three experimental groups: group 1 (sham) received vehicle injection of medium only (n = 5); group 2 transplanted with EMBs (n = 4); and group 3 transplanted with fresh RMBs (n = 5). All animals received the same volume of fluid.

#### Measurement of liver function tests and creatinine

Assessment the severity of hepatic damage after D-GalN-induce ALF was performed on blood samples collected on day 1, 2, 4 and 8 post-ALF induction compared to day 0 (baseline control). Prothrombin time (PT), ammonia, alanine aminotransferase (ALT), aspartate aminotransferase (AST), bilirubin and creatinine were analysed at the same time point. PT and ammonia were determined using fresh whole blood without anticoagulants (8 µl and 20 µl, respectively). PT was measured using an automated coagulation monitoring device (CoaguCheck XS System, Roche Diagnostic, Mannheim, Germany) with a maximum recorded value of >96 seconds. Ammonia was determined using a blood ammonia analyser (PocketChem BA, Manarini Diagnostics, Wokingham, UK). The measurement range of this test is 7–286 µmol/L. ALT, AST, bilirubin and creatinine were measured in serum samples (150 µl) using a routine biochemical AutoAnalyser (Advia 2400; Siemen Healthcare Diagnostics, Camberley, UK).

#### Animal follow-up and rat hepatocyte microbeads retrieval

Survival rate was followed for 7 days post transplantation. Animals were euthanised at day 7 or if any sign of distress was observed. The signs of distress were marked respiratory depression with cyanosis, rapid weight loss >30% over 24 h, significant bleeding, and marked neurological signs as a consequence of liver failure which are typically agitation followed by a lack of response to external stimuli and animals becoming immobile. Microbeads and liver tissue were harvested from the abdomen by laparotomy. To retrieve microbeads, the peritoneal cavity was flushed several times with 50 ml CMRL followed by thorough inspection of the abdominal cavity for signs of inflammation or adhesion.

#### Evaluation of retrieved rat hepatocyte microbeads and liver histology

Retrieved microbeads were examined for morphology, including host cell adhesion to their surface to determine the extent of host immune responses. Cell viability and function of retrieved microbeads were evaluated after maintenance in culture for 24 h after retrieval. Histopathology analysis was performed on liver tissue samples.

### Statistical analysis

Data were analysed using GraphPad Prism 6 software (GraphPad, CA, USA). All data are presented as the mean±standard error of the mean (mean±SEM) from at least 4 independent experiments unless otherwise stated. Student *t*-test was applied to compare two independent groups. A two-way measurement ANOVA and Turkey's multiple comparisons test were used to compare groups exposed to different conditions. Survival curves were calculated using the Kaplan-Meier methods and compared with log-rank test. A p-value of ≤0.05 was considered statistically significant.

## Results

### Effects of polymerisation time on physical integrity and stability of HMBs

HMBs in all three control groups were uniform with a mean diameter of 584±0.9 µm (10 min), 582.2±1.2 µm (15 min), and 584.4±1.2 µm (20 min), with no statistical differences between the three polymerisation groups. There was a significant increase in mean microbeads 'diameter in all samples incubated in hypotonic solution compared to both control and CMRL groups (p<0.001). The 15 min polymerisation HMBs group had a significantly better mechanical stability compared to the other two groups (p<0.001) with a mean microbead diameter difference of 7.1 µm (10 min vs 15 min), and 23.5 µm (20 min vs 15 min) after exposure to water.

### Effects of polymerisation time on hepatocyte viability and function in HMBs

There was no statistically significant difference in cell viability (MTT) between the three polymerisation times for HMBs under the same conditions. MTT activity was maintained after 3 h incubation in CMRL compared to control, however, in all groups there was a marked decrease in cell activity when incubated in water (p<0.02). HMBs from the three polymerisation groups provided similar metabolic function after being maintained in culture for 24 h. The amount of albumin production for 10, 15 and 20 mins group were 569.0±112.2, 606.3±97.0, and 581.2±103.3 ng/mg protein and urea production were 6.1±1.1, 6.1±1.2, and 6.4±1.2 µg/mg protein, respectively.

### Effects of cell density on microbead morphology, human hepatocyte viability and functions

**Figure 1:**
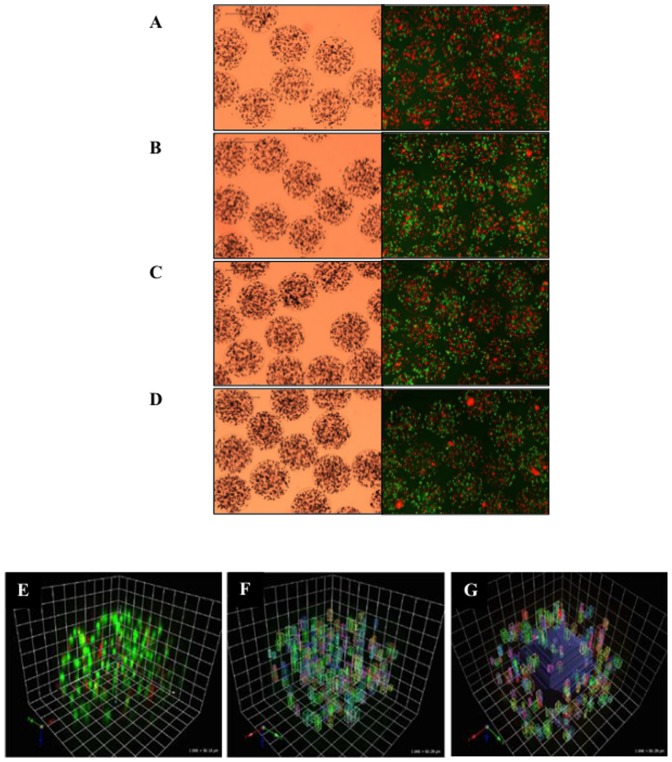
Distribution of human hepatocytes in alginate-hepatocyte microbeads. Representative image of HMBs produced with different cell densities; 2.0, 2.5, 3.0 and 3.5×10^6^ cells/ml alginate (A, B, C, and D respectively) under light microscopy (left) and fluorescence microscopy (right). Representative confocal microscopy images used in 3D reconstruction to demonstrate (E & F) cell distribution and viability across the microbead, and (G) viability of cells within outer half vs inner half of same microbead. Green; viable and red; dead cells.

**Figure 2:**
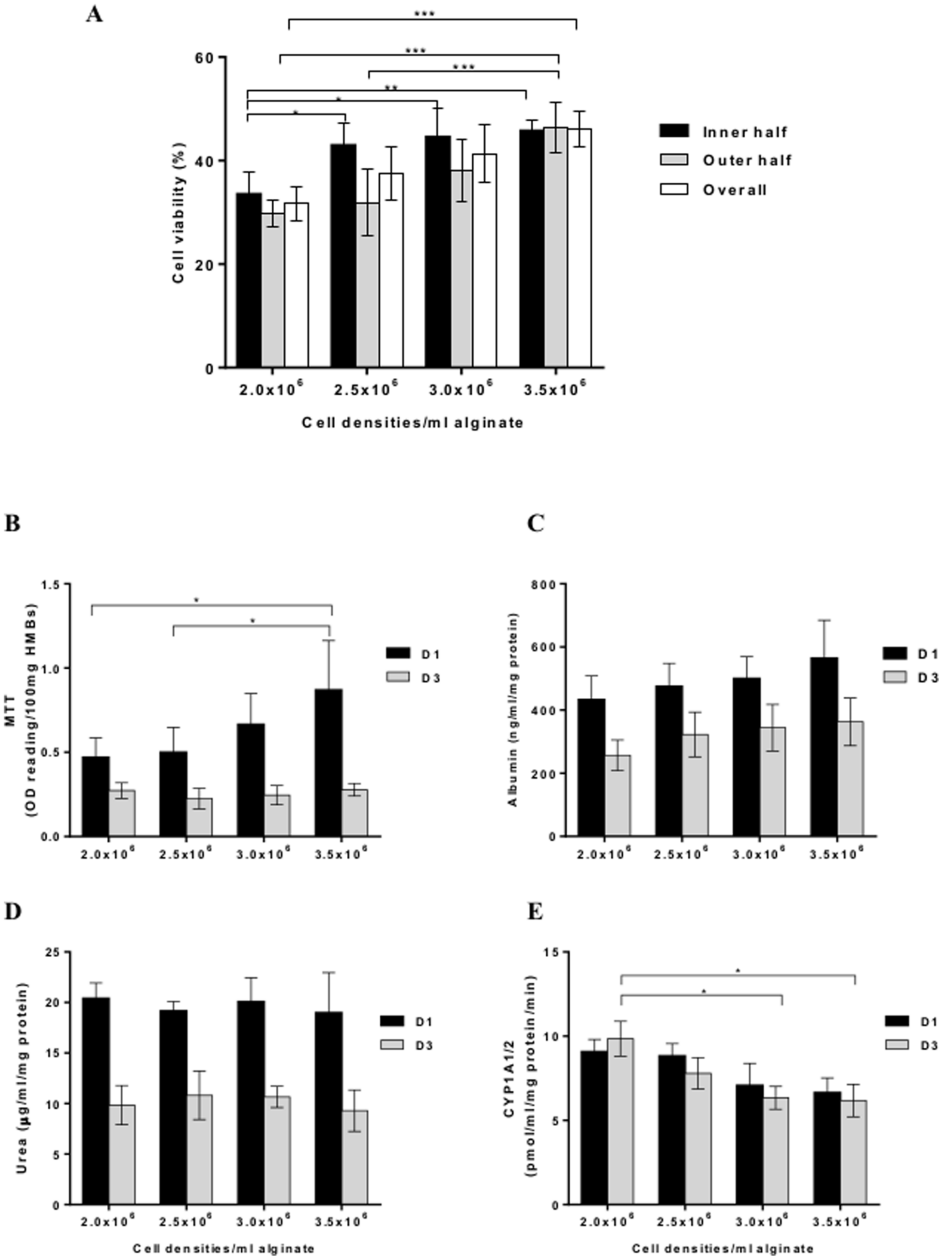
Effect of cell density on cell viability and human hepatocyte function. Cell viability demonstrated by (A) 3D reconstruction technique and (B) MTT activity. Hepatocyte functions measured by (C) albumin production, (D) urea production and (E) CYP1A1/2 activity; *p<0.05, **p≤0.01, ***p≤0.001.

The viability of human hepatocytes before encapsulation was 61.5±1.1%. HMBs produced using different cell densities (2.0×10^6^ to 3.5× 10^6^ cells/ml alginate) showed uniformity and a smooth outer surface with no significant differences between all groups. After encapsulation, HMBs were maintained in culture overnight to allow the encapsulated hepatocytes to stabilise. Cell viability (FDA/PI staining) showed that the density of 3.5×10^6^ cells/ml provided the highest viability. The 3D reconstructed images showed that cells were evenly distributed inside the microbead (Figure 1). The average cell number per microbead for each cell density ranged from 201 (2.0×10^6^ cells/ml alginate) up to 447 (3.5×10^6^ cells/ml alginate). Surprisingly, as the cell density was increased there was no significant difference in cell viability between outer and inner halves in all groups (Figure 2A). The overall viability in the 3.5×10^6^ cells/ml group was significantly higher than 2.0×10^6^ cells/ml (46.1±3.3% vs 31.7±3.3%, p = 0.01) and also tended to be higher than 2.5×10^6^ cells/ml (37.42±5.17%) but did not reach significance (p = 0.06). Furthermore, MTT assay on day 1 showed a similar trend where the 3.5×10^6^ cells/ml group had a higher activity than other groups. However, on day 3 there was a significant decrease in overall activity for 3.0×10^6^ and 3.5×10^6^ cells/ml (p<0.05; Figure 2B).

Urea and albumin assays showed no significant differences between the 4 groups on either day 1 or day 3 (Figures 2C and D). However, albumin synthesis showed a similar pattern to cell viability, where the higher cell number gave higher production. By contrast, the CYP1A1/2 activity on day 3 of the 2.0×10^6^ cells/ml group was significantly higher than 3.0 and 3.5×10^6^ cells/ml (p<0.05; Figure 2E).

### Cell viability and function in HMBs after maintenance in ascitic fluid

**Figure 3:**
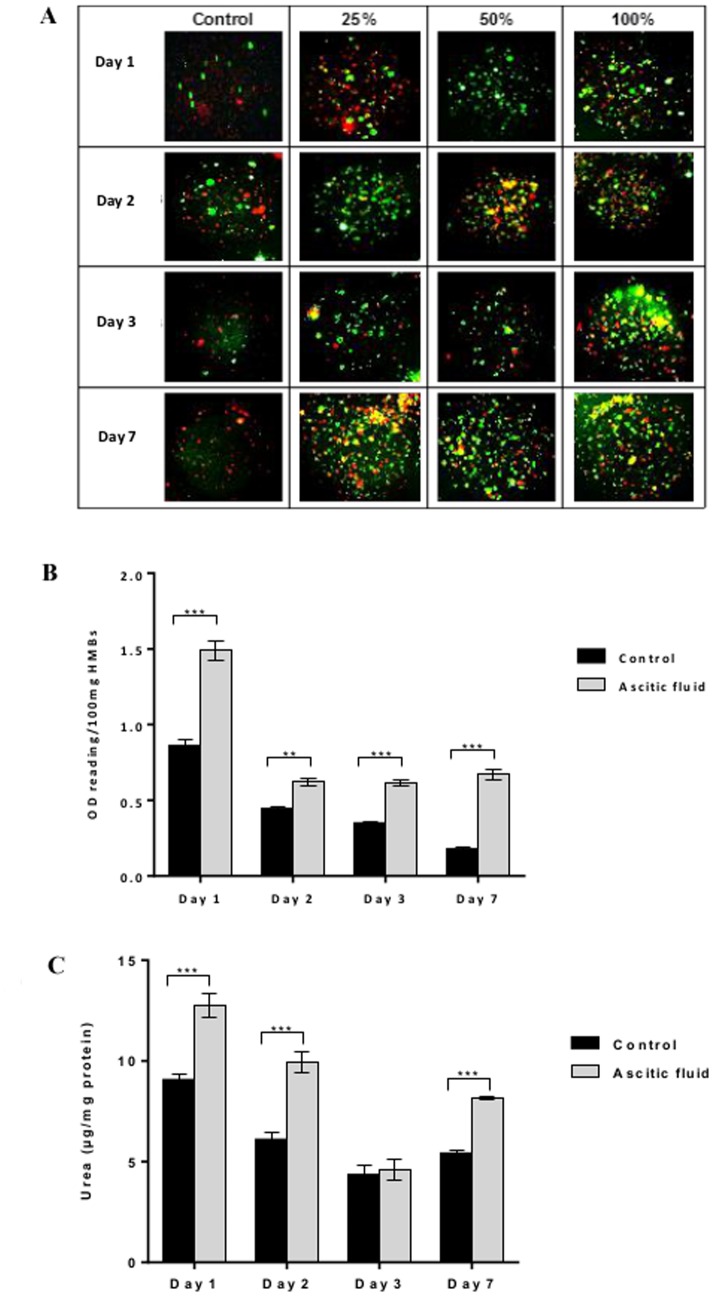
Effect of ascitic fluid on human hepatocyte microbeads (HMBs) cell viability and activity. (A) FDA/PI staining of HMBs maintained in supplemented culture medium or ascitic fluid at different concentrations (25%, 50%, and 100%), (B) MTT assay and (C) urea production compared between HMBs incubated in supplemented culture medium and ascitic fluid, respectively; *P<0.05, **P≤0.01, ***P≤0.001.

Microbeads cultured in ascitic fluid showed higher cell viability (FDA/PI staining) than those in supplemented culture medium (controls) at all-time points (Figure 3A). Similarly, overall cell activity (MTT) of HMBs cultured in ascitic fluid was significantly higher than controls (p<0.001) on day 1 (OD reading/100 mg HMBs: 1.49±0.07 vs. 0.86±0.04) up to day 7 (0.67±0.03 vs. 0.18±0.02) of culture (Figure 3B). Moreover, urea production showed higher levels in HMBs incubated in ascitic fluid compared to controls (p<0.001) at all times except on day 3, and on day 7 urea level was significantly higher (6.3±0.2 vs 3.6±0.4 µg/mg protein, p<0.001), (Figure 3C).

### Activation level of PBMCs co-cultured with EMBs and HMBs

After co-culturing microbeads with PBMCs for 24 h, both EMBs and HMBs were still intact and maintained their uniform shape without any apparent PBMCs adherent to their surface. The percentage of frequency of activation marker expression on PBMCs is shown in Table 1. There were no significant differences in the levels of overall cell activation markers expression (CD3^+^CD38^+^, CD4^+^CD38^+^, CD8^+^CD38^+^) on PBMCs co-cultured with EMBs or HMBs compared to PBMCs in monoculture (control). Also, the percentage of CD4^+^CD25^+^, CD3^+^CD25^+^, CD8^+^CD25^+^ T cells expression did not differ between the PBMCs co-cultured with microbeads (EMBs or HMBs) and control. The frequency of CD3^+^C56^+^ NK cells; CD19^+^CD25^+^and CD19^+^CD54^+^ B cells were also comparable between the three groups. The frequency of CD14^+^CD25^+^ activated monocytes in PBMCs co-cultured with HMBs was significantly lower compared to those co-cultured with EMBs, and PBMCs monocultures (2.66±0.8% vs 22.8±7.7% and 45.7±11.0%, respectively; p<0.0001).

### *In Vitro* studies of RMBs

**Figure 4:**
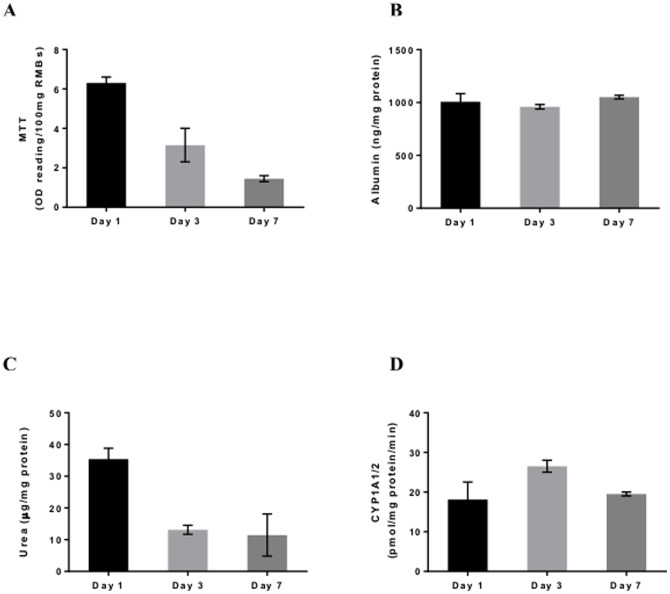
Cell viability and metabolic functions of optimised rat hepatocyte microbeads (RMBs). (A) Cell viability showed by MTT assay, (B) albumin production, (C) urea production and (D) CYP1A1/2 activity.

The viability of rat hepatocytes used for encapsulation was >70%. After microencapsulation using the optimised technique, the RMBs obtained were of uniform shape with viability in the MTT assay of 7.65±1.26 OD reading/100 mg microbeads. Over the period of 1 week in culture, although cell viability of RMBs progressively decreased, hepatocyte-specific functions including albumin production and CYP1A1/2 activity was remained stable. Urea production was decreased on day 3 but then maintained until day 7 (Figure 4).

### *In Vivo* studies

D-GalN at 1.2 g/kg induced ALF defined by PT>36 sec (INR≥3) at 24 h after ALF induction in about 50% of rats. Liver function of ALF-rats was blood ammonia; median 21 (range 7-58) µmol/l, PT; 66.5 (37.4–96.0) sec, AST; 2260 (1017–6255) IU/l, ALT; 1218 (803–5584) IU/l, and bilirubin; 3 (2–10) µmol/l.

After intraperitoneal transplantation, all animals in the three groups immediately recovered from the injection without any post-operative complications or mortality and became ambulatory. Subsequently, two animals in group 2, transplanted with EMBs, died on day 2 and day 3 post-ALF induction. In group 1, one animal died on day 3. All animals in group 3 transplanted with RMBs survived for 1 week post transplantation. The 72-hour survival rates were 80%, 50%, and 100% in group 1, 2 and 3, respectively, after this time the survival rate did not change in all experimental groups The rats in group 3 (treated with RMBs) had better survival than the other two groups (untreated and treated with EMBs); but this was no statistically significant.

**Figure 5:**
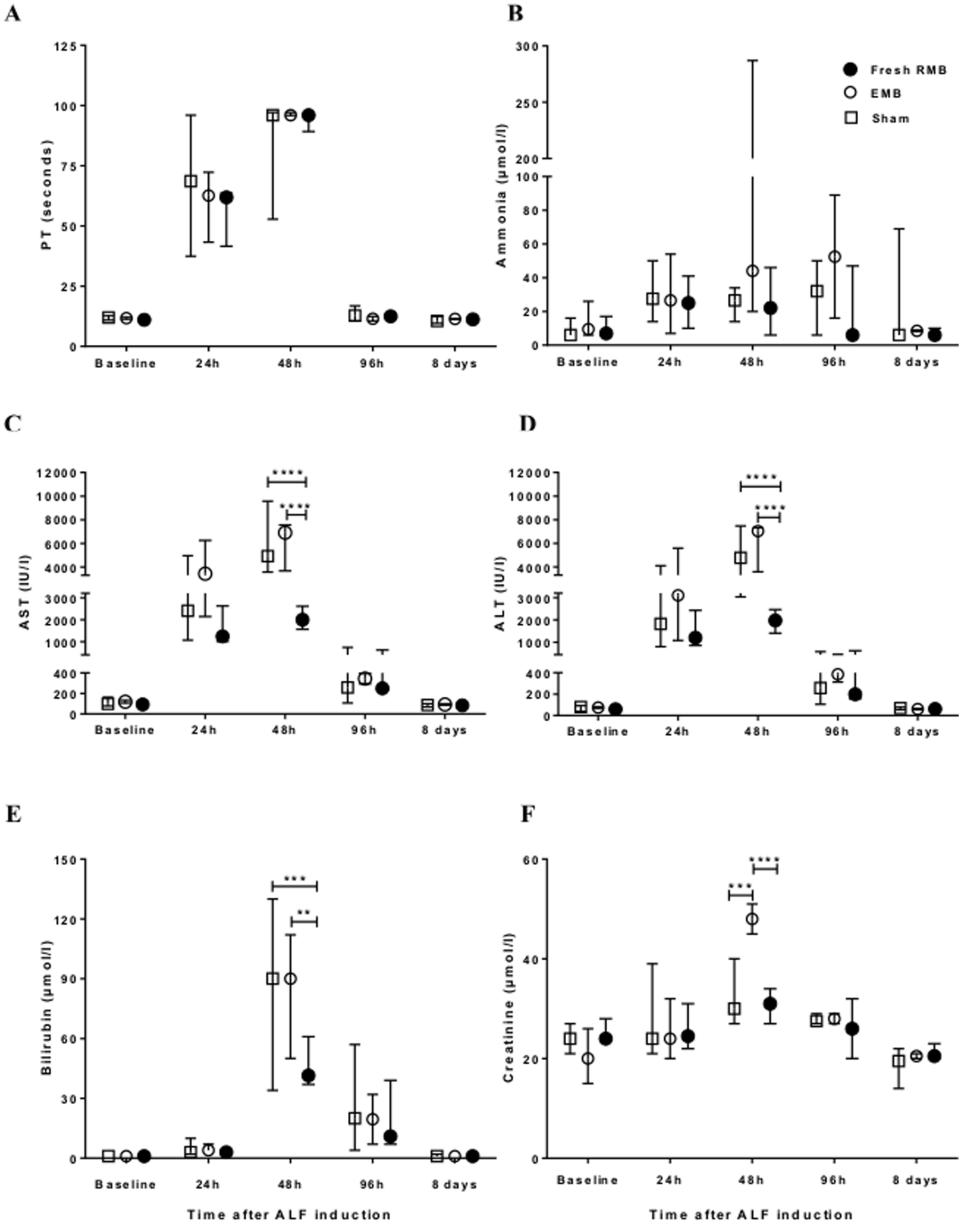
Effect of intraperitoneal transplantation of rat hepatocyte microbeads on biochemical indexes of liver injury. Liver injury was evaluated by (A) prothrombin time (PT), (B) blood ammonia level, (C) serum aspartate aminotransferase (AST) level, (D) serum alanine aminotransferase (ALT) level, (E) serum bilirubin level and renal impairment assessed by (F) serum creatinine. Data are expressed as median and range; statistical significance*p<0.05, **p<0.01, ***p<0.001 and ****p<0.0001.

The blood chemistry results started to increase from baseline levels significantly by 24 h and then progressively rose to reach a peak at 48 h after ALF induction indicating the point of maximal liver injury. A general improvement in liver function was observed in surviving animals on day 4 post-ALF induction. Subsequently, all parameters returned to normal levels (baseline) on day 8 post-ALF induction (Figure 5). There was no significant difference in biochemical profiles at 24 h after ALF induction with a similar degree of liver injury in all groups before receiving microbead treatment. The differences between groups were clearly seen on the following day after transplantation of microbeads. In the rats in group 3, transplantation with RMBs reduced the severity of ALF with a statistically significant lower level of ALT, AST and bilirubin than those of group 1 (p<0.0001, p<0.0001 and p<0.001, respectively) and group 2 (p<0.0001, p<0.0001, and p <0.01, respectively). In addition, the creatinine level in rats transplanted with RMBs (24.5, range 22–31 umol/l) was significantly lower than in those transplanted with EMBs group (48, range 45–51 umol/l; p<0.01). PT and ammonia levels were not significantly different between the 3 groups.

**Figure 6:**
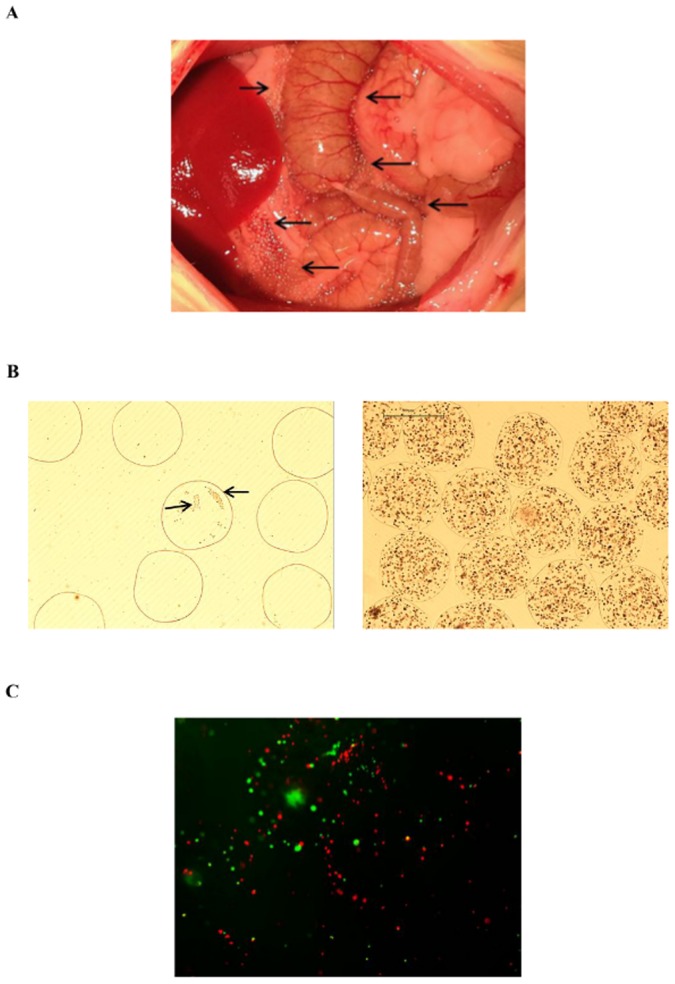
Intraperitoneal transplantation of rat hepatocyte microbeads in rats with ALF. Representative images of (A) microbeads 7 days post-transplantation distributed throughout abdominal cavity [black arrow] without any signs of inflammation or adhesion; and (B) recovered microbeads, EMBs (left) partly covered by host cells [black arrow] and RMBs (right) with no evidence of cell adhesion; and (C) viable cells in RMBs demonstrated by FDA/PI staining.

Microbeads were recovered from surviving animals 7 days after transplantation or whenever the animals died (or were euthanised). Microbeads were found dispersed throughout the peritoneal cavity mainly loosely attached to omentum and mesentery, which came off easily after flushing with transplant medium. No signs of inflammation or adhesion were observed (Figure 6A). The total volume of capsules retrieved was found to be 70±2.4% of those transplanted. The retrieved microbeads maintained their integrity (Figure 6B). There was hardly any host cell adherent on the surface of retrieved microbeads (≤1%). The retrieved RMBs in group 3 showed viable cells as demonstrated by FDA/PI staining (Figure 6C) and MTT activity (0.7±0.1 OD reading/100 mg RMBs). Hepatocyte function for albumin synthesis (1033.3±22.4 ng/mg protein), urea synthesis (10±5.5 µg/mg protein) and CYP1A1/2 activity (9.9±0.1 pmol/mg protein/min) in culture was also seen.

**Figure 7:**
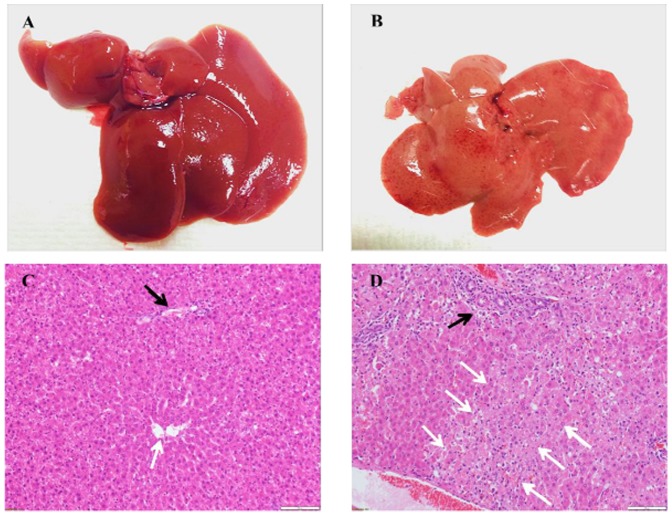
Livers of rats with ALF. Representative image of gross appearance of liver; (A) surviving rats at day 7 post transplantation showed normal appearance as normal control rats, and (B) Rats that died at day 2 after D-GalN injection appeared yellowish pale colour, and atrophy. Representative histopathology sections of liver tissue from rats with D-GalN induced ALF; H&E staining (200x), scale bars = 100 µm. (C) surviving rat at 7 days post transplantation showed normal lobular architecture, and (D) rat that died at 48 h after ALF induction showed confluent hepatocellular necrosis affecting centrilobular region; portal tract [black arrow] and central lobular region [white arrow].

Liver histopathology of surviving rats showed completely normal intact lobular architecture without signs of hepatocellular injury 1 week post transplantation. ALF rats that died showed evidence of severe hepatocellular necrosis affecting periportal and centrilobular region (Figure 7).

## Discussion

The “ideal” microbeads must have physical stability and biocompatibility without compromising the microenvironment inside the microbead to allow long-term cell survival and function. After exposure to osmotic stress, HMBs demonstrated that a polymerisation time of 15 min resulted in more physically stable microbeads compared to the other two groups. The likely reason for this is that 10 min polymerisation is too short for effective cross-linking and equilibration of external Ca^2+^ between the core of alginate and the external medium [21], whereas an increase in polymerisation time to 20 min made microbeads become brittle [22], [23]. Our results showed that a prolonged polymerisation time did not have a negative effect on encapsulated cell viability and function, supporting previous studies [22], [24].

We investigated the influence of cell density on physical integrity, cell viability and function in order to establish the optimal ratio of cell mass per microbead to allow oxygen, nutrients, and metabolite diffusion. The results showed that microbeads produced using these cell densities 2.0×10^6^, 2.5×10^6^, 3.0×10^6^, and 3.5×10^6^ cells/ml, all had a stable physical integrity with a smooth surface, but when the cell density was increased to 4.0×10^6^ cells/ml alginate, surface irregularities and microbead deformities were observed. These findings are different from a recently published study by Durkurt and colleagues (2013) who suggested that for rat hepatocytes the optimal density is 1.0×10^6^ cells/ml alginate [25]. An increase in microbead cell content results in a considerable change in the microenvironment inside the microbeads with the possibility of apoptotic and hypoxic cell death particularly towards the microbead centre. It is difficult to assess the cells while they are entrapped in the three-dimensional structure. Therefore, we determined the cell number, distribution, and viability across microbeads using both fluorescent (2D) and confocal (3D) microscopy. It has been reported that encapsulated hepatocytes lose approximately 20–30% viability after encapsulation [26]. In this study, cell viability in HMBs was assessed after maintenance in culture for 1 day instead of immediately after preparation to allow stabilisation of cells to the microenvironment. We found that 3.5×10^6^ cells/ml alginate HMBs provided the highest cell viability with an overall viability loss after encapsulation of 24%. It must be noted that, the cell viability loss could be attributed to quality of the available hepatocytes used, due to steatosis and cryopreservation injury. In order to improve hepatocyte function and viability, high quality fresh cells should be used. We are also investigating other methods to improve the viability of hepatocytes such as co-encapsulation with mesenchymal stem/stromal cells [27]. Another possibility could be to improve the hepatocyte function by using short-activating RNA oligonucleotides to enhance liver-specific gene expression [28]. The 3D confocal microscopy data showed that cell viability in the centre of the microbead did not decrease as the cell number per microbead was increased. The gel network of Ca-alginate is an inhomogeneous structure in which the alginate and calcium concentrations are lower in the core than the outer part of the microbead [23], thus allowing oxygen, nutrients, and metabolites to penetrate the microbead. Albumin and urea production tended to be better in the higher cell density groups but this did not reach statistical significance. CYP1A1/2 activity was, however, lower in the high cell density than the low cell density groups. Optimisation of the polymerisation time and cell density has contributed to the improvement in efficiency of the HMBs.

We also evaluated this optimised technique for encapsulation of rat hepatocytes. The RMBs produced showed similar properties to the HMBs with good morphology and well maintained functionality.

We tested the microbeads in ascitic fluid which mimics the microenvironment around the HMBs following intraperitoneal transplantation. The data obtained showed that HMBs maintained their *in vitro* viability and functions in ascitic fluid better than in normal culture medium. This could be explained by the microenvironment characteristics of peritoneal fluid which contains a variety of soluble factors such as growth factors, cytokines, steroids, angiogenic factors and trace elements [29], [30].

Animal studies have shown that host immune and inflammatory reactions towards microbeads (allogenic and xenogenic cells) after intraperitoneal transplantation could lead to host cell adhesion to the microbead surface. Hepatocyte microbeads survived and functioned for up to two weeks, but some cells died in the centre of the microbead [4], [31]. To our knowledge none of the published studies have investigated alloimmune reaction against human hepatocyte microbeads. Therefore, we tested the immunogenicity of GMP grade HMBs. Our findings demonstrated that both EMBs and HMBs did not elicit immune activation *in vitro*. This is probably due to ultrapure sodium alginate being used and is consistent with other studies testing inflammation and immune reaction related to the purity of alginate [12], [32]. Rokstad et al (2011) showed that alginate microbeads are complement compatible [33].

We have also performed preliminary experiments to assess the suitability and function of optimised HMBs in normal rats. Human albumin was detected in the serum of rats for the 7 days of study. Explanted microbeads were intact and were minimally covered with adherent host cells, the hepatocytes were viable, and able to synthesise albumin and urea in culture (unpublished data). We transplanted RMBs in rats with ALF so that fresh cells could be used due to the limited and timely availability of fresh human hepatocytes. This also avoided any possible xenogeneic reactions using human hepatocytes in rats and may better simulate the transplantation of HMBs in patients. Our study showed a positive outcome for intraperitoneal transplantation of optimised microbeads in ALF-rats. The technique was safe without any immediate or late complications. The hepatocytes inside the microbeads survived and were able to function well without using immunosuppression. Rats with transplantation of RMBs all survived, which tended to be better than with EMBs and untreated animals, however, it did not reach statistical significance due to the small numbers. Also the lower mortality of ALF with D-GalN at this dose [34] made it difficult to assess. However, there was a significant improvement in biochemical parameters including ALT, AST, and bilirubin of RMBs groups compared to the other two groups. We also evaluated serum creatinine to determine the severity of ALF and multiorgan failure and found that the RMBs group had significantly lower creatinine than EMB group. The liver histology of surviving rats showed complete recovery after 7 days of transplantation. Our data suggests that intraperitoneal transplantation of hepatocytes microbeads containing only a small part of the total liver mass could support the failing liver and improve the clinical outcome. Umehara et al showed that transplanting encapsulated cells at 4% of total liver volume improved metabolism and survival rate of ALF rats [4], whereas others transplanted up to 30% of liver mass [31], [35], [36]. However, the optimal cell number required to provide efficient function in the clinical setting of ALF remains controversial and further studies are needed.

Transplantation of the optimised RMBs *in vivo* also showed encouraging results in terms of the physical integrity and biocompatibility of hepatocyte microbeads and their survival and function. The retrieved microbeads were intact and maintained their shape and size after 1 week in the peritoneal cavity. Most of microbeads were free from any host cell adhesion. Previous studies reported transplanted microcapsules (sodium alginate-poly-L-lysine-sodium alginate copolymer) surrounded by fibrous tissue within 4 days to 2 weeks [4], [37]. In addition, in our study there was no evidence of fibrosis or tissue adhesion surrounding the microbeads. This could be due to the clinical grade alginate we used, which does not provoke the host immune response in keeping with our *in vitro* data. The viability and the detection of albumin and urea production, and cytochrome activity produced by the retrieved RMBs support on-going microbead function after transplantation.

Currently, a pilot study of treatment of children with acute liver failure by intraperitoneal transplantation of HMBs is being performed at our centre. Encapsulation of fresh hepatocytes followed by cryopreservation to establish a good quality HMBs bank is important for the future. A study to develop protocols for cryopreservation of hepatocyte microbeads is underway. This will lead to microbeads being readily available for immediate use in patients with ALF.

In conclusion, an optimised protocol for production of GMP grade human hepatocyte microbeads has been established. The alginate encapsulated cells maintained their hepatocyte-specific function *in vitro* and *in vivo*. Intraperitoneal transplantation of optimised RMBs provided metabolic support in ALF without immunosuppression and without complications. These high quality alginate human hepatocyte microbeads should be suitable for use in clinical transplantation.
