# Correction: Alginate Microencapsulated Hepatocytes Optimised for Transplantation in Acute Liver Failure

**DOI:** 10.1371/journal.pone.0119226

**Published:** 2015-03-18

**Authors:** The PLOS ONE Staff

RRM was omitted from a grant in the Funding section. The correct Funding information is: This work was supported by the National Institute for Health Research (RRM, AD, RDH and NDH), funds for consumables and WellChild (RRM and AD), funds were given to purchase the encapsulator used in the work. The funders had no role in study design, data collection and analysis, decision to publish, or preparation of the manuscript.
